# Epithelial-derived cytokines in the pathogenesis of severe asthma

**DOI:** 10.3389/falgy.2025.1681147

**Published:** 2025-10-15

**Authors:** Duong Duc Pham, Tae-Bum Kim

**Affiliations:** Department of Allergy and Clinical Immunology, Asan Medical Center, University of Ulsan College of Medicine, Seoul, Republic of Korea

**Keywords:** severe asthma, epithelial-derived cytokines, alarmin, type 2 inflammation, non-T2 asthma

## Abstract

**Introduction:**

Airway epithelial cells function as the first physical barrier against pathogens and are key regulators of immune responses by producing a wide array of cytokines involved in both innate and adaptive immunity.

**Methods:**

This review summarizes recent advances in our understanding of epithelial-derived cytokines in severe asthma (SA) pathogenesis and highlights promising therapeutic strategies.

**Results:**

Epithelial-derived cytokines can be functionally classified into the following four main groups: alarmins [interleukin [IL]-25, IL-33, thymic stromal lymphopoietin [TSLP]], proinflammatory cytokines (IL-1, IL-6, tumor necrosis factor-α), chemokines (CCL2, CCL5), and antiviral cytokines [interferon (IFN)-α, IFN-β, IFN-λ]. Alarmins are rapidly released in response to epithelial injury and play a pivotal role in initiating immune responses by activating dendritic cells, type 2 innate lymphoid cells, and eosinophils. Proinflammatory cytokines intensify inflammation by promoting immune cell activation and cytokine cascades, while chemokines guide immune cells to sites of injury. Antiviral cytokines enhance epithelial defenses by inducing the expression of antiviral genes. In SA, epithelial-derived cytokines play a central role in initiating and sustaining type 2 (T2) inflammation by activating the IL-4, IL-5, and IL-13 axis, leading to increased eosinophils, elevated serum IgE, and heightened airway hyperresponsiveness. These cytokines are also implicated in non-T2 inflammation, particularly in refractory asthma phenotypes.

**Discussion:**

Growing insights into epithelial cytokines and their complex signaling networks with the airway microenvironment have opened new avenues for developing targeted and personalized treatment in SA.

## Introduction

Airway epithelial cells form the first line of defense against inhaled pathogens and environmental toxins, serving both physical and immunological barrier functions (1). These cells develop, maintain, and repair the respiratory tract by producing mucus, regulating inflammation, and facilitating tissue remodeling (2). Beyond their structural role, epithelial cells are actively involved in innate immunity (3). Through pattern recognition receptors, including Toll-like receptors, NOD-like receptors, and RIG-I-like receptors, airway epithelial cells detect microbial components and initiate intracellular signaling cascades (4). In response, they secrete a variety of antimicrobial peptides, such as defensins, cathelicidins, and lysozymes, which directly contribute to the elimination of pathogens (5). Epithelial cells also release cytokines and chemokines that direct immune cell recruitment and activation (3). Following injury, airway epithelial cells rapidly proliferate and differentiate to restore barrier integrity and prevent secondary infections (6).

Asthma is a chronic inflammatory disease of the airways characterized by variable respiratory symptoms, including wheezing, shortness of breath, chest tightness, cough, and expiratory airflow limitation (7). Severe asthma (SA), affecting approximately 5%–10% of patients with asthma, is a complex clinical condition characterized by persistent symptoms that remain uncontrolled despite adhering to guideline-recommended therapy (8). SA encompasses multiple inflammatory phenotypes, including type 2 (T2) inflammation with elevated IL-4, IL-5, and IL-13 driving eosinophilia, IgE production, and airway hyperresponsiveness (9), as well as non-T2 phenotypes such as neutrophilic airway inflammation, obesity-associated asthma, and paucigranulocytic asthma (10). Recent studies have highlighted the importance of airway epithelial cell-derived cytokines in SA (6, 11). These cytokines activate both innate and adaptive immune responses, amplifying inflammation and perpetuating chronic disease. In this review, we systematically summarize the latest findings on the role of epithelial-derived cytokines in SA, focusing on their immunological function and potential as therapeutic targets.

## Airway epithelium as a physical barrier

**Figure 1:**
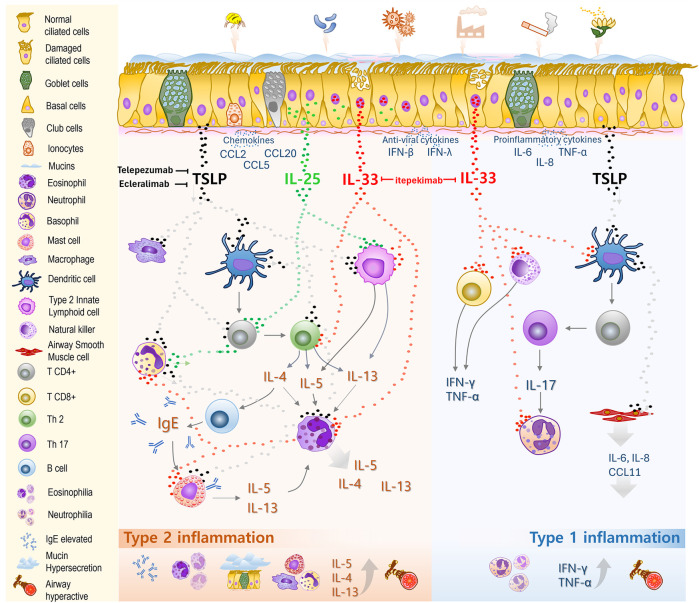
Roles of epithelial-derived cytokines in the pathogenesis of severe asthma. The airway epithelial barrier comprises ciliated and basal cells, along with secretory cells such as goblet cells, club cells, and ionocytes, all covered by a mucus layer propelled by ciliary motion. This forms the first line of defense against inhaled allergens, pollutants, and pathogens. Upon exposure, these agents trigger the release of various cytokines from epithelial cells. Epithelial-derived alarmins, thymic stromal lymphopoietin (TSLP), interleukin (IL)-25, and IL-33, play key roles as initiators and amplifiers of inflammation. TSLP drives dendritic cells (DCs) to induce CD4^+^ *T* cell differentiation into Th2 cells, enhances IgG and IgE production, activates ILC2s and basophils, and stimulates the proliferation and differentiation of mast cells and macrophages toward a type 2 inflammatory response. TSLP indirectly promotes Th17 differentiation, leading to IL-17 production and neutrophilic inflammation, driving a type 1 inflammatory response. It also stimulates airway smooth muscle cells to release proinflammatory cytokines. IL-33, stored in the nucleus and released upon cellular or tissue damage, acts on both type 2 (Th2 cells, ILC2s, eosinophils) and type 1 (CD8^+^ *T* cells, neutrophils) immune cells, thereby bridging and amplifying both inflammatory pathways. IL-25, normally stored in the cytoplasm of epithelial cells, primarily targets T2 immune cells such as ILC2s, Th2 cells, and basophils, boosting T2 inflammatory responses. Together, these cytokine pathways drive airway inflammation, mucus hypersecretion, bronchoconstriction, and airway remodeling in severe asthma. Targeting epithelial-derived cytokines has become a promising therapeutic approach, with tezepelumab (anti-TSLP) already approved for clinical use.

The airway epithelial barrier comprises epithelial cells interconnected by adhesion proteins, such as zonula occludens-1, occludin, and claudins, which form tight junctions that establish the first line of defense against airborne environmental threats, including house dust mites, pollen, and pollutants (12, 13). This barrier comprises three key cell types: ciliated cells, which coordinate mucociliary clearance by propelling mucus to remove inhaled particles and pathogens; basal cells, located at the base of the epithelium, which function as progenitor cells to regenerate damaged or senescent epithelial cells; and secretory cells (such as goblet cells), which produce mucins to form the protective mucus layer, and in some cases, cytokines (11, 14) (Figure 1).

In asthma, the airway epithelial barrier can be disrupted by the breakdown of tight junctions, leading to increased permeability to pathogens and environmental allergens (15, 16). This disruption is observed across all asthma phenotypes. In severe T2-high asthma, epithelial barrier dysfunction is associated with FcεRI–IgE cross-linking, which induces activation of Src family kinases, alarmin expression, loss of junctional proteins, and increased epithelial permeability (17).

Mucus hypersecretion is common in asthma, particularly SA. The mucus obstructs the airways and leads to uncontrolled airway inflammation and recurrent exacerbations (18). In SA, overactivation of the IL-4/IL-13 pathway drives excessive mucus production **(**19). Additionally, eosinophils release eosinophil peroxidase (EPO), which promotes the formation of dense and highly viscous mucins, further impairing airflow (20). Goblet cell hyperplasia, combined with a reduction in the number and function of ciliated epithelial cells, worsens mucus retention (21).

Basal cells serve as the primary progenitors of the airway epithelium, playing a crucial role in tissue regeneration. They maintain epithelial homeostasis by proliferating and differentiating to replenish specialized cells, such as goblet and ciliated cells after injury (11, 21). However, in SA, airway epithelial cells, including basal cells, exhibit markedly increased proliferation compared to those in mild asthma or healthy individuals, potentially contributing to airway remodeling (22). Moreover, structural damage to the epithelium may trigger the release of growth factors such as TGF-β, which impairs the ability of basal cells to regenerate and perpetuates epithelial dysfunction (23).

Club cells (also known as Clara cells) are non-ciliated epithelial cells located in the bronchioles. They secrete anti-inflammatory proteins (club cell secretory protein/CC10), secrete protective proteins and surfactant components, detoxify inhaled substances via cytochrome P450 enzymes, and support epithelial regeneration after injury (24). Ionocytes are rare, specialized epithelial cells characterized by high expression of cystic fibrosis transmembrane conductance regulator, a chloride channel essential for ion transport and regulation of airway surface liquid (ASL). This function maintains mucosal hydration and effectively supports mucociliary clearance (25, 26).

Beyond structural cells, innate immune cells, including dendritic cells (DCs) and mast cells, are scattered within or adjacent to the airway epithelium. Although they do not directly contribute to the physical barrier, they are critical in recognizing environmental stimuli and activating other innate immune cells as well as adaptive immune responses, thereby protecting the airways from harmful agents.

## Immune functions of airway epithelial cells

In addition to forming a physical barrier, airway epithelial cells also play a central role in orchestrating immune responses by releasing a wide range of cytokines. Epithelial-derived cytokines are functionally grouped into four categories: alarmins, proinflammatory cytokines, chemokines, and antiviral cytokines. Alarmins play a pivotal role in airway immune responses in patients with severe asthma and will be discussed in detail in the following sections.

Following injury or IL-13 stimulation, epithelial cells—particularly basal cells and goblet cells—upregulate the secretion of proinflammatory cytokines such as IL-6, IL-8, and TNF-α via NF-κB and AP-1 signaling pathways (27–29), thereby recruiting inflammatory cells, amplifying inflammatory responses, and promoting remodeling through the release of growth factors (30, 31).

Chemokines are small (∼15 kDa) signaling molecules secreted by epithelial cells that guide the migration of immune cells to sites of inflammation (32). According to their structural motifs, chemokines are classified into two main families (CC and CXC) and two subgroups (C and CX3C) (33). CC chemokines primarily attract monocytes, lymphocytes, dendritic cells, eosinophils, and basophils, but not neutrophils (34). Viral or bacterial infections induce the release of epithelial chemokines, including CCL2, CCL5, and CCL20 (35), while house dust mite allergens trigger thymus- and activation-regulated chemokine, enhancing Th2 cell recruitment (36). In asthma, CCL2 is often overexpressed, and blocking CCL2 or its receptor alleviates symptoms (37). CC chemokines such as CCL11, CCL24, CCL26, and CCL5 recruit eosinophils to allergic sites and activate them via CCR3 signaling, promoting inflammation and the release of intracellular granules (38–40). Meanwhile, CXC chemokines predominantly recruit neutrophils and other inflammatory cells (34). The association of CXCL5 with its receptor CXCR2 has been linked to eosinophilia during asthma exacerbations (41). Additionally, the CXCL10/CXCR3 axis is involved in the recruitment of mast cells to inflamed regions, contributing to the contraction of airway smooth muscle (ASM) cells in asthma (42).

The airway epithelium produces type I interferons, particularly IFN-β, in response to viral infections. IFN-β enhances antiviral defenses by inducing interferon-stimulated genes and recruiting NK, CD4+, and CD8+ *T* cells (43). IFN-β deficiency is associated with asthma severity, and clinical trials with inhaled IFN-β have shown reduced asthma exacerbations caused by viruses (44). The mechanisms underlying IFN-β deficiency in asthma and the therapeutic role of epithelial cytokines require further investigation (45). Due to the functional diversity and complex interactions, non-alarmin epithelial-derived cytokines present considerable difficulties in pinpointing precise therapeutic targets. The understanding of the role of non-alarmin cytokines in SA remains limited. To date, no large-scale studies have demonstrated significant success in developing targeted treatments in this field.

## Epithelial alarmins: initiators and boosters of inflammation

The key alarmins include TSLP, IL-25, and IL-33. Their release may occur concurrently or selectively, depending on the nature of the environmental insult.

### TSLP: the spark of inflammation

TSLP, a member of the IL-2 cytokine family, was first isolated from thymic stromal cell cultures and initially linked to B cell development (46). Structurally composed of four α-helical bundles, TSLP is encoded by the *TSLP* gene on chromosome 5q22. It signals through a heterodimeric receptor comprising the TSLP receptor chain (TSLPR) and the IL-7 receptor alpha chain (IL-7Rα) (47). In humans, TSLP exists in two isoforms: the short form (sfTSLP) and the long form (lfTSLP), each with distinct functions (48). sfTSLP is constitutively expressed in healthy barrier tissues such as the epithelium of the lungs, gut, and skin, contributing to immune homeostasis and displaying potential anti-inflammatory and antimicrobial functions by suppressing pro-inflammatory cytokines such as TNF-α, IL-1β, and IL-6 (49). In contrast, lfTSLP is induced by inflammatory stimuli, including allergens, infections, and cytokines, and strongly promotes type 2 (T2) inflammatory responses (50). Recent studies have demonstrated that lfTSLP disrupts airway epithelial barrier function, whereas sfTSLP can counteract these detrimental effects (51). Additionally, lfTSLP has been shown to induce autophagy in ASM cells, thereby promoting airway inflammation and remodeling in both *in vitro* and *in vivo* asthma models, while sfTSLP exerts inhibitory effects on these processes (52).

In the lungs, TSLP is primarily secreted by epithelial cells but is also produced by fibroblasts, DCs, basophils, and mast cells (53). TSLP secretion is triggered by various factors, including PAR-2-activating proteases (e.g., airborne fungi like Alternaria) (54), dsRNA from viruses such as rhinovirus or RSV (55), and uric acid from house dust mites (56). There are several mechanisms of TSLP-mediated immune activation. TSLP acts on DCs, promoting their ability to stimulate naive CD4+ *T* cells to differentiate into Th2 cells, which produce T2 cytokines such as IL-4, IL-5, and IL-13 (57). TSLP may also directly influence CD4+ *T* cells to differentiate into T follicular helper cells, enhancing the production of IgG and IgE (58). TSLP inhibits regulatory *T* cells, removing negative regulation and allowing unchecked T2 inflammation (59, 60). TSLP and IL-33 jointly activate group 2 innate lymphoid cells, leading to IL-5 and IL-13 production (61). TSLP also promotes basophil production independently of IL-3 (62, 63), enhances the expression of CD203c, supports eotaxin-mediated migration, and forms an IL-3 autocrine loop, contributing to allergic airway inflammation (64).

When stimulated by TNF-α, the expression of TSLPR and TSLPR mRNA increases, activating eosinophils and the release of eosinophil-derived neurotoxin (65). In bronchial epithelial cells, TSLP delays apoptosis, enhances fibronectin adhesion, and induces secretion of IL-6, IL-8, CXCL1, and CXCL2 (66). TSLP also promotes the proliferation and differentiation of mast cells from bone marrow by activating signal transducer and activator of transcription 6 (STAT6) and upregulating mouse double minute 2 homolog (MDM2), intensifying allergic inflammation (67). TSLP drives the differentiation of macrophages into an alternatively activated T2 inflammatory phenotype (68).

TSLP also contributes to non-type 2 (non-T2) inflammatory processes, including non-allergic asthma and non-eosinophilic asthma. In the context of viral infections or non-allergic triggers, TSLP-stimulated DCs promote the differentiation of naive CD4+ *T* cells into Th2 cells and promote Th17 differentiation, particularly in the presence of IL-1β, IL-6, and IL-23 (69). These Th17 cells secrete IL-17, which contributes to the recruitment of neutrophils and induces neutrophilic inflammation, a hallmark of non-T2 asthma (10). TSLP also activates ASM cells, enhancing secretion of IL-6, CXCL8 (IL-8), and CCL11 (eotaxin-1) (70).

Clinically, elevated TSLP levels in bronchoalveolar lavage fluid and airway tissues correlate with disease severity and airway obstruction in children with asthma (71). *TSLP* gene expression is increased in the airway epithelium and mucosa of adult patients with SA (72), and high plasma TSLP levels correlate with persistent asthma exacerbations (73). In SA, TSLP has been implicated in corticosteroid resistance, through regulation of STAT5 phosphorylation and Bcl-xL expression in natural helper cells (74). Beyond T2 inflammation, TSLP promotes airway remodeling, by inducing bronchial smooth muscle cell migration and proliferation, contributing to bronchial wall thickening and remodeling in chronic asthma (75). In mouse models of chronic asthma, TSLP inhibition improved airway remodeling features, such as peribronchial collagen deposition and goblet cell hyperplasia (76).

### IL-25: amplifier of T2-inflammation

IL-25, also known as IL-17E, is a member of the IL-17 cytokine family encoded on chromosome 14 (77). IL-25 is produced by airway epithelial cells (78), intestines, and colon, and is also produced by Th2 cells, alveolar macrophages (79), eosinophils, basophils (80), and mast cells (81). IL-25 signals through a heterodimeric receptor composed of IL-17RA and IL-17RB (also known as IL-17Rh1 or EVI27), both of which need to be present to activate downstream signaling pathways (82). Under normal conditions, IL-25 is continuously produced and stored in the cytoplasm of resting normal human bronchial epithelial cells and secreted upon exposure to allergen-derived proteases (83).

IL-25 mRNA expression increases upon exposure to airborne allergens (78, 84). IL-25 directly promotes the differentiation of CD4^+^ *T* cells into Th2 cells, via the production of cytokines such as IL-4, IL-5, and IL-13, which are central to T2 inflammation. This process depends on the initial autocrine production of IL-4 by T cells and the presence of the signaling protein STAT6 (78). IL-25 enhances Th2 cytokine production, recruits eosinophils and CD4^+^ T cells to the airways, and induces goblet cell hyperplasia, which are hallmarks of allergic airway inflammation (78, 85). IL-25 also upregulates eotaxin and arginase-1, two key factors in recruiting eosinophils into lung tissue, thereby amplifying the T2 inflammatory response (86).

IL-25 is a potent activator of group 2 innate lymphoid cells (ILC2), which are immune cells lacking specific antigen receptors but capable of producing type 2 inflammatory cytokines such as IL-5, IL-9, and IL-13 (87). Activated ILC2 promotes mucus secretion, bronchoconstriction (88), and the recruitment of eosinophils and mast cells, which enhances the inflammatory response (89, 90). This cytokine loop supports IgE production, further fueling allergic type 2 inflammation (91). The initiation and amplification of type 2 inflammation via ILC2 also involves other alarmins such as TSLP and IL-33. Furthermore, IL-25 directly enhances antigen uptake in eosinophils and activates Th2 cells in allergic inflammation (92). IL-25 promotes eosinophil activation and migration (93), while reducing apoptosis (94), thereby amplifying type 2 inflammation. Unlike TSLP and IL-33, IL-25 is currently not associated with non-T2 inflammation (10, 95), reinforcing its role as a T2-specific amplifier.

In asthma, IL-25 is elevated (96), particularly during rhinovirus-triggered exacerbations (97). High IL-25 levels, even in the absence of TSLP or IL-33, correlate with greater T2 inflammation and increased responsiveness to inhaled corticosteroids (96). IL-25 expression in the sputum of asthma patients is associated with asthma severity (98). Evidence suggests that IL-25 contributes to airway remodeling in SA by inducing a fibrotic phenotype shift in airway epithelial cells and circulating fibroblasts (99), promoting fibroblast proliferation, extracellular matrix deposition (100), and a fibrotic epithelial phenotype.

### IL-33: tissue damage alarm

IL-33 is a member of the IL-1 cytokine family, acting as a vital alarmin that helps maintain tissue balance and repair while participating in both type 1 and type 2 immune responses (101). IL-33 is encoded on chromosome 9p24.1 (102). IL-33 is predominantly expressed in the nucleus of epithelial, endothelial, and mesenchymal cells, including basal cells of the airway epithelium (103). IL-33 is stored in the nucleus and can be released in response to proteases derived from allergens or pathogens (104–106), during cellular stress (107), or in the course of apoptosis (108, 109).

IL-33 is synthesized and stored in the nucleus as a full-length form (IL-33-FL), which has modest biological activity. Upon cellular stress or necrosis, IL-33-FL is released into the extracellular space. Its biological fate then depends on the proteases involved: (i) proteolytic processing by allergen-derived or endogenous proteases (e.g., elastase, cathepsin G, chymase, or tryptase) generates mature IL-33 forms, which are up to 60-fold more potent and bind with high affinity to its receptor ST2, promoting a rapid type 2 inflammatory response (110); (ii) conversely, cleavage by apoptotic caspases produces inactive IL-33 fragments, thereby preventing inappropriate immune activation (108).

ST2 is also known as IL-1 receptor-like 1 (IL1RL1) (111) and exists in two forms: the membrane-anchored form (ST2l) and the soluble form (sST2) (112). ST2l mediates IL-33 signaling, while sST2 acts as a “decoy” receptor binding to IL-33 in the extracellular fluid to prevent it from interacting with ST2l, thereby regulating or inhibiting IL-33 signaling (113). Upon IL-33 binding, ST2l associates with a co-receptor, IL-1 receptor accessory protein (IL-1RAcP). The formation of the IL-33/ST2l/IL-1RAcP complex activates intracellular signaling pathways such as nuclear factor kappa-light-chain-enhancer of activated B cells (NF-κB) and mitogen-activated protein kinase, leading to T2 cytokine production (114).

IL-33 impacts the immune system and tissue repair through various mechanisms. In type 2 inflammation, IL-33 activates Th2 cells via the transcription factor GATA3, promoting the production of IL-5 and IL-13 (115, 116). The IL-33/ST2 signaling axis activates ILC2, increasing the production of IL-5 and IL-13 (117), and collaborates with TSLP to phosphorylate STAT5, amplifying type 2 inflammation (118). For eosinophils, IL-33 stimulates degranulation, enhances superoxide production similar to IL-5, prolongs cell survival, and increases adhesion capacity (119, 120). In basophils, IL-33 upregulates the surface expression of the CD11b antigen, enhances adhesion, promotes migration to inflammatory sites (121), and increases IgE-dependent degranulation (122), reinforcing its role in T2 inflammation. IL-33 also stimulates mast cell differentiation, releasing cytokines (IL-5, IL-6, IL-10, and IL-13) and chemokines (CCL1, CXCL8), contributing to allergic inflammation (123).

Respiratory infections, such as viral (e.g., rhinovirus) or bacterial (e.g., influenza) infections, are key factors that trigger and aggravate exacerbations in patients with non-T2 SA (124). IL-33 drives non-T2 inflammation to clear these pathogens, potentially worsening airway inflammation. IL-33 cooperates with IL-12 and TCR signaling to increase IFN-γ production in CD8+ *T* cells, promoting viral clearance and CD8+ *T* cell differentiation (125, 126). It also stimulates DCs to produce IL-6 and upregulate surface molecules, thereby driving type 1 inflammation via TNF-α and IFN-γ (127), and contributes to neutrophil activation in patients with uncontrolled SA (128).

In ASM cells, IL-33 aids wound healing through IL-13 from mast cells (129). IL-33 also increases collagen and fibronectin production in fibroblasts, contributing to airway remodeling in asthma (130, 131). Since IL-33 is released upon cellular or tissue damage, initiating inflammatory and tissue repair responses, it is considered a “tissue damage alarmin.”

Clinically, IL-33 and ST2 levels are elevated in the serum and sputum of asthma patients and correlate with disease severity (132, 133). IL-33 expression is markedly elevated in ASM cells from endobronchial biopsy samples of patients with SA (134). Unlike TSLP, IL-33 plays a key role in driving virus-induced exacerbations in asthma patients (135).

## Alarmin-targeted therapies for SA

Recent advances in SA treatment have coincided with improved phenotypic classification, particularly the recognition of T2 inflammation (9). The development of monoclonal antibodies targeting T2 pathways, such as anti-IL5 or anti-IL4/IL13, has substantially alleviated the burden of SA (136). However, a subset of patients, especially those lacking T2 inflammatory biomarkers, do not respond to these therapies (10). In this context, targeting TSLP, a cytokine that initiates inflammatory responses upon allergen exposure, may provide therapeutic benefits for various SA phenotypes, including those that do not respond to T2-targeted biologic therapies.

Tezepelumab, a fully humanized IgG2λ monoclonal antibody targeting TSLP, is currently the only successful anti-TSLP therapy (137–139). Approved for commercial use since 2021, it is indicated for the treatment of uncontrolled SA in patients aged 12 and older and administered via subcutaneous injection (140). Tezepelumab significantly reduces asthma exacerbation rates according to two major clinical trials. In the PATHWAY trial (phase 2), patients receiving tezepelumab experienced a 62%–71% reduction in annual exacerbation rates compared to placebo, alongside improvements in lung function, with efficacy independent of baseline eosinophil counts **(**138). In the NAVIGATOR trial (phase 3), tezepelumab reduced exacerbation rates by 56% compared to placebo, including in patients with elevated blood eosinophils, while also improving lung function, asthma control, quality of life, and symptom severity (139). To further reduce systemic side effects and improve safety, inhaled anti-TSLP products, such as ecleralimab, are being developed. Ecleralimab (CSJ117), an inhaled anti-TSLP antibody fragment, binds to soluble TSLP and prevents TSLP receptor activation (141). Phase IIa studies indicate that ecleralimab can reduce bronchoconstriction and airway inflammation caused by allergens in patients with mild allergic asthma (142).

Monoclonal antibodies targeting IL-25 (anti-IL-25) (143) or its receptor IL-17RB (144, 145) remain in preclinical development or early research stages. Studies in mice indicate that IL-25 inhibition can prevent airway hyperresponsiveness, reduce the production of T2 inflammation-related cytokines, decrease eosinophil infiltration, limit goblet cell hyperplasia, and lower serum IgE levels (146). Anti-IL-25 agents, originally developed for treating respiratory viral infections, may also help manage severe asthma exacerbations associated with viral infections (143). Despite promising findings, no anti-IL-25 agents have advanced to phase II clinical trials.

The IL-33/ST2 axis is under investigation as a therapeutic target for patients with SA, particularly those who have not responded to previous biologic therapies. Several monoclonal antibodies in trials include GSK3772847, REGN3500 (itepekimab), and ANB020 (etokimab). A phase IIa trial of GSK3772847 in patients with moderate to SA and allergic fungal airway disease showed no significant efficacy, possibly due to a small sample size (147). Itepekimab, an anti-IL-33 monoclonal antibody, reduced exacerbation rates and improved lung function compared to placebo in patients with moderate to SA but did not provide superior benefits compared to dupilumab (148). A phase 2b trial of etokimab in patients with severe eosinophilic asthma has been completed, but official results have not yet been published (149). Overall, drugs targeting the IL-33/IL1RL1 axis hold potential as alternatives to current type 2 monoclonal antibodies, but the efficacy of combining these therapies requires further investigation.

## Conclusion

Airway epithelial cells play a central role in asthma by secreting proinflammatory cytokines, chemokines, antiviral cytokines, and alarmins, driving diverse inflammatory responses, including T2 and non-T2 inflammation. Understanding their mechanisms of action and interaction networks offers opportunities for targeted therapies in SA, particularly for non-T2 phenotypes. Among current treatments, tezepelumab (a commercialized anti-TSLP agent) has demonstrated efficacy in reducing exacerbation rates and improving lung function, while therapies targeting IL-25 and IL-33 show promise in preclinical studies. However, clinical data on IL-25 and IL-33 remain limited, and large-scale studies on non-alarmin epithelial cytokines are lacking. Future research should focus on cytokine-specific pathways, evaluating combination therapies, and developing inhaled biologics to optimize efficacy and minimize systemic side effects. Large-scale clinical trials targeting non-T2 phenotypes and epithelial-driven airway remodeling pathways are essential to advance personalized asthma treatments.
